# Skin Mycetoma in an 11-Year-Old African Boy: Case Presentation with Emphasis on Histopathological Features and Differential Diagnosis

**DOI:** 10.3390/dermatopathology8040053

**Published:** 2021-10-26

**Authors:** Gerardo Cazzato, Anna Colagrande, Antonietta Cimmino, Lucia Lospalluti, Aurora Demarco, Caterina Foti, Paolo Romita, Francesca Arezzo, Vera Loizzi, Paola Parente, Leonardo Resta, Giuseppe Ingravallo

**Affiliations:** 1 Section of Pathology, Department of Emergency and Organ Transplantation (DETO), University of Bari Aldo Moro, 70124 Bari, Italy; anna.colagrande@gmail.com (A.C.); micasucci@inwind.it (A.C.); leonardo.resta@uniba.it (L.R.); 2 Section of Dermatology, Department of Biomedical Sciences and Human Oncology, University of Bari Aldo Moro, Piazza Giulio Cesare 11, 70124 Bari, Italy; l.lospalluti@gmail.com (L.L.); aurorademarco94@gmail.com (A.D.); caterina.foti@uniba.it (C.F.); paolo.romita@uniba.it (P.R.); 3 Section of Ginecology and Obstetrics, Department of Biomedical Sciences and Human Oncology, University of Bari Aldo Moro, Piazza Giulio Cesare 11, 70124 Bari, Italy; francesca.arezzo@uniba.it (F.A.); vera.loizzi@uniba.it (V.L.); 4 Pathology Unit, Fondazione IRCCS Casa Sollievo della Sofferenza, 71013 San Giovanni Rotondo, Italy; paolaparente77@gmail.com

**Keywords:** mycetoma, skin, actinomycetes

## Abstract

Mycetoma is an uncommon, chronic infective disease of the skin and subcutaneous tissues, characterized by the triad of tumefaction, draining sinuses, and the presence in the exudate of colonial grains. In cases of long-term disease, the presence of colonial grains together with the host’s derivative material can lead to the formation of real sinuses. Histological analysis is of fundamental importance to allow an accurate etiological diagnosis and to understand if the basic pathogen is an actinomycete (bacterium) or a real fungus (eumycetic mycetomas) and is also fundamental for therapy, which is quite different. Here, we present a case of Mycetoma in an 11-year-old patient who emigrated from Djibouti, Somalia, and showed the essential histopathological features of this rare and forgotten nosographic entity in the industrialized world and briefly discuss the major and most important differential diagnoses.

## 1. Introduction

The first description of mycetomas dates back to the Sanskrit religious work “Atharva Veda” (2000–1000 BC) in which an infirmity called “padavalmika” (anthill foot) is mentioned. The first “modern” description is due to Gill, who, in 1842, reported the observation in the district of Madurai of some cases located in the lower limbs (hence the denomination of “foot of Madura” and “maduromycosis”), even if the more complete presentation was due, a few years later (1844–1845), to Godfrey Garrison, a surgeon in Madras who described four cases. We owe to Carter, in 1860, the description of the infectious character of the mycetoma, with his “degenerative theory” of the “black grains” from the “white ones” described in the monograph “On mycetoma or the fungus disease of India”, in which there is a description of all the clinical and etiological aspects with a very precise description of *Madurella mycetomatis*. There are many synonyms of mycetoma—foot of Madura, maduromycosis, disease of Godfrey and Eire, morbus tuberculosis pedis, morbus pedis endophyticus, endemic degeneration of the bones and foot, perforating ulcer of the foot—even if the only recognized names are: mycetoma and foot of Madura [1].

Mycetoma is an infectious disease characterized by the presence of a generally slow-evolving granuloma. There are two main etiological groups of mycetoma: actinomycetic mycetomas, which are caused by aerobic filamentous bacteria of the order *Actinomycetales* [2,3], and eumycetic (maduromycotic) mycetomas caused by a number of species of true fungi [4]. In cases of long-term disease, the presence of colonial grains together with the host’s derivative material can lead to the formation of real sinuses (6–12 months) [5]. The histological analysis is of fundamental importance to allow an accurate etiological diagnosis and to understand the basic pathogen, considering the microscopic histological characteristics of the various pathogens. Indeed, the size of the grains varies from microscopic to 1–2 mm in diameter; for example, large grains are observed with *madurellae* and with *Actinomadura madurae*, whereas the granules of *Nocardia brasiliensis* and *N. asteroides* are small [6]. Even the colors of the grains are different; for example, dark (black) grain mycetomas are found only among the eumycetic mycetomas (melanoprotein or related substance). While the consistency of most grains is soft, those of *Streptomyces somaliensis* and *Madurella mycetomatis* can be quite hard [7,8].

The body districts usually involved are the acral parts of the limbs, but sometimes also other sites, and, in addition, extension to the skin and subcutis, muscles, and bones is common [9]. Rarely there is lymphatic dissemination to regional lymph nodes [10].

## 2. Case Presentation

**Figure 1:**
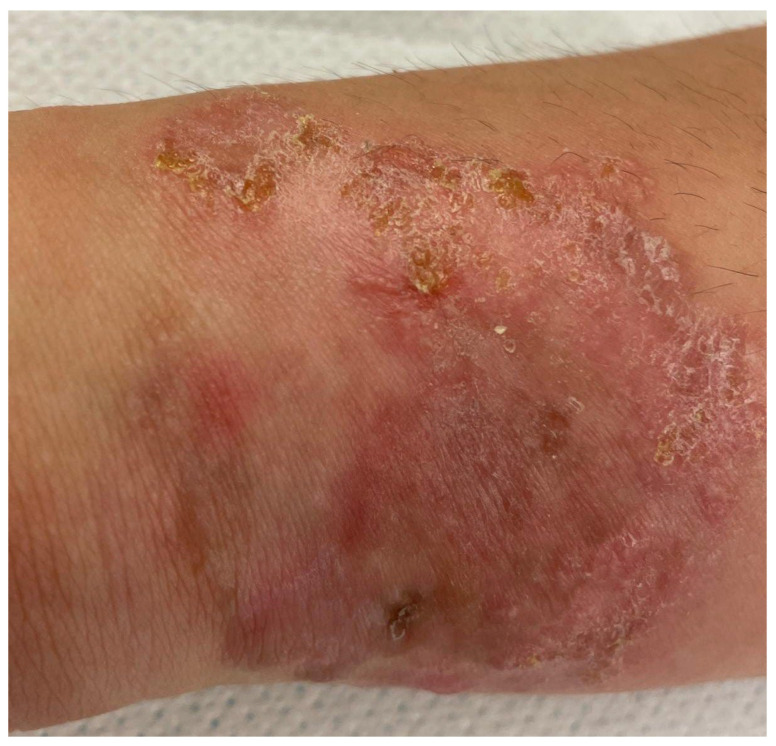
Figured lesion with clear margins and irregular edges, predominantly bright red erythema with the presence of crusty, scaly elements.

An 11-year-old boy came from Djibouti, Somalia, with unspecified lesions affecting the malleolar and peri-malleolar region of the right foot. He reported no significant symptoms. He had come to the doctor’s attention, after arriving in Italy, for an accidental trauma to the other foot, which, however, did not show any noteworthy injury. During the inspection, a clinical situation such as that shown in Figure 1 was described, with presence of a figured lesion with clear margins and irregular edges, predominantly bright red erythema with the presence of crusty, scaly elements. Due to the absence of significant symptoms, the patient had never presented at any hospital, although he complained, when questioned, of a certain difficulty in walking (running gear) and putting on socks.

**Figure 2:**
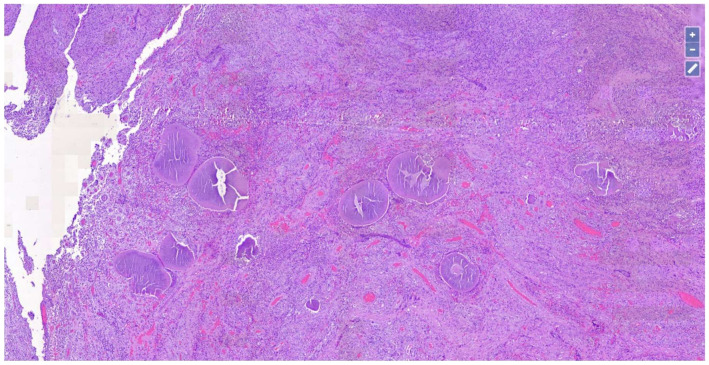
Chronic inflammatory infiltration of giant cell epithelioid-granulomatous around haematoxylinophilic material consisting of grains and host organic substances (Hematoxylin-Eosin, Original Magnification: 4×).

**Figure 3:**
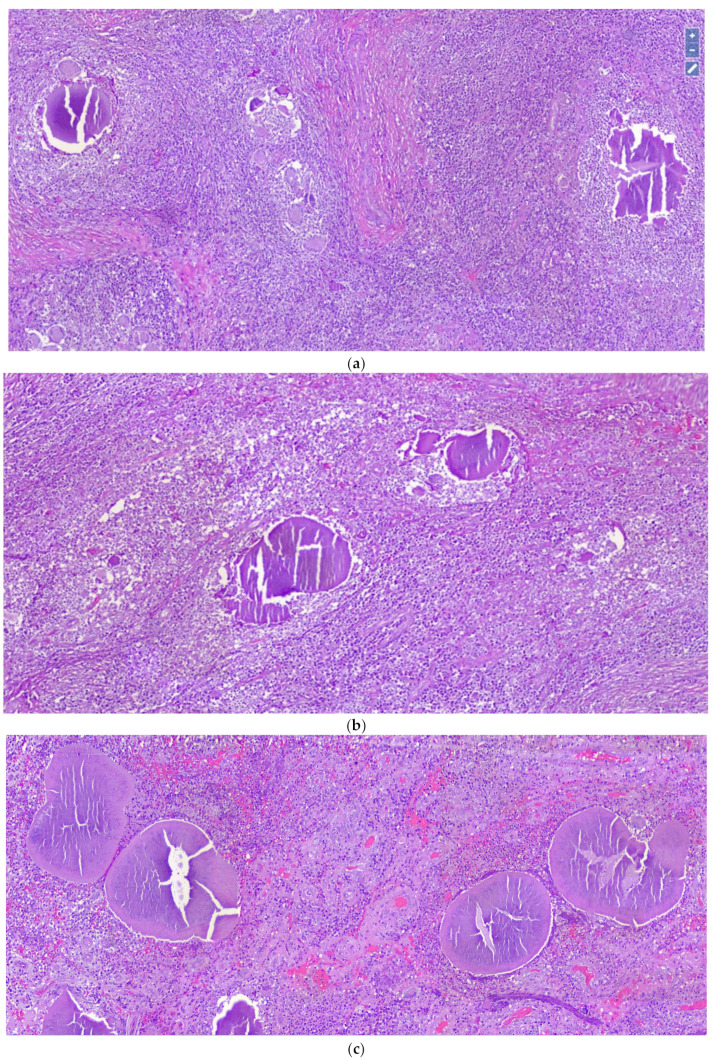
(**a**) Histological detail of the previous image (Hematoxylin-Eosin, Original Magnification: 10×). (**b**,**c**) Note large grains with dense thin filaments, often stains homogeneously, with transverse fracture lines (Hematoxylin-Eosin, Original Magnification: 20×).

Upon histopathological observation, the lesion consisted of a dense and diffuse gigantocellular epithelioid-granulomatous inflammatory infiltrate (Figure 2), in the dermal and subcutaneous area, arranged around grains (0.5–2 mm, with dense thin filaments, stains homogeneously and with transverse fracture lines) and organic substances of the host (Figure 3). Furthermore, there was a neutrophilic granulocytic inflammatory infiltrate, sometimes in abscess evolution, and moderate fibrosis phenomena.

Microbiological culture tests confirmed *Streptomyces somaliensis* infection, and therapy with Amikacin in combination with Trimethoprim-sulfamethoxazole was initiated promptly. After about 3 weeks of therapy, the lesions began to respond and shrink in diameter.

Unfortunately, we were unable to carry out further immunohistochemical investigations because the patient returned to Somalia with the block of his material.

## 3. Discussion

Although mycetoma is a disease of tropical countries, particularly West Africa, parts of India, and Central and South America, there are sporadic reports of cases in the USA, Canada, and Europe, where new migratory flows are modifying the epidemiology observed and known until a few years ago [11,12]. In these countries, rural workers, particularly males, are most commonly infected, with over 70% of infections occurring on the feet (Madura foot), with the hand the next most common site of involvement [13]. The grains discharged from the sinuses vary in color, size, and consistency, features that are used for the identification of mycetoma [14]. For example, black grains are characteristic of *Madurella Mycetomatis* in Eumycetomas, while pale grains are present in infection with *Pseudoallescheria boydii* or *Aspergillus nidulans*.

Instead, in cases of actinomycetoma, red grains are present in the case of infection with *Actinomadura pelletieri*; yellow grains in the case of *Streptomyces somaliensis* infection (as in our case described), and pale grains in the case of *Nocardia brasiliensis*, *N. otitidiscaviarum*, *N. asteroids*, and *Actinomadura madurae* [6,15,16,17,18].

In terms of size, the large segmented mycelial filaments (2–4 micrometer in diameter, with club-shaped hyphal swellings and chlamydospores) are characteristic of the true fungi that cause eumycetomas in contrast with the Gram-positive thin filaments (1 micrometer or less) of the organism that cause actinomycetomas [19,20].

For instituting a proper patient treatment plan, accurate identification of the causative organism is vital. For actinomycetoma, different laboratory-based techniques have been developed during recent decades. These include direct microscopy, cytology, histopathology, and serology. More recently, different molecular techniques and matrix-assisted laser desorption ionization-time of flight mass spectrometry have been included as diagnostic methods for actinomycetoma [21,22,23].
