# Correction: Cazzato et al. Skin Mycetoma in an 11-Year-Old African Boy: Case Presentation with Emphasis on Histopathological Features and Differential Diagnosis. *Dermatopathology* 2021, *8*, 509–514

**DOI:** 10.3390/dermatopathology13020021

**Published:** 2026-05-08

**Authors:** Gerardo Cazzato, Anna Colagrande, Antonietta Cimmino, Lucia Lospalluti, Aurora Demarco, Caterina Foti, Paolo Romita, Francesca Arezzo, Vera Loizzi, Paola Parente, Leonardo Resta, Giuseppe Ingravallo

**Affiliations:** 1 Section of Pathology, Department of Emergency and Organ Transplantation (DETO), University of Bari Aldo Moro, 70124 Bari, Italy; anna.colagrande@gmail.com (A.C.); micasucci@inwind.it (A.C.); leonardo.resta@uniba.it (L.R.); 2 Section of Dermatology, Department of Biomedical Sciences and Human Oncology, University of Bari Aldo Moro, Piazza Giulio Cesare 11, 70124 Bari, Italy; l.lospalluti@gmail.com (L.L.); aurorademarco94@gmail.com (A.D.); caterina.foti@uniba.it (C.F.); paolo.romita@uniba.it (P.R.); 3 Section of Ginecology and Obstetrics, Department of Biomedical Sciences and Human Oncology, University of Bari Aldo Moro, Piazza Giulio Cesare 11, 70124 Bari, Italy; francesca.arezzo@uniba.it (F.A.); vera.loizzi@uniba.it (V.L.); 4 Pathology Unit, Fondazione IRCCS Casa Sollievo della Sofferenza, 71013 San Giovanni Rotondo, Italy; paolaparente77@gmail.com

The authors would like to make the following corrections to this published paper [1].

In the original publication, there was a mistake in Figure 1 as published. Figure 1 was mistakenly included in the manuscript as it was believed to be original due to a miscommunication among the authors. The corrected Figure 1 appears below.

To prevent any misunderstandings and ensure clarity, the authors would like to revise the description in Section 2. “Case Presentation” from:

“During the inspection, a clinical situation such as that shown in Figure 1 was described, with a presence of diffuse nodular lesions, extensively affecting the cutaneous and subcutaneous plane, of variegated color (from yellowish to dark red), with hardened and multiple base vesicles subcutaneous abscesses, which, after rupture, resulted in real superficial skin sinuses. Due to the absence of significant symptoms, the patient had never presented at any hospital, although he complained, when questioned, of a certain difficulty in walking (running gear) and putting on socks”

To:

“During the inspection, a clinical situation such as that shown in Figure 1 was described, with presence of a figured lesion with clear margins and irregular edges, predominantly bright red erythema with the presence of crusty, scaly elements. Due to the absence of significant symptoms, the patient had never presented at any hospital, although he complained, when questioned, of a certain difficulty in walking (running gear) and putting on socks”.

The authors state that the scientific conclusions are unaffected. This correction was approved by the Academic Editor. The original publication has also been updated.

## Figures and Tables

**Figure 1:**
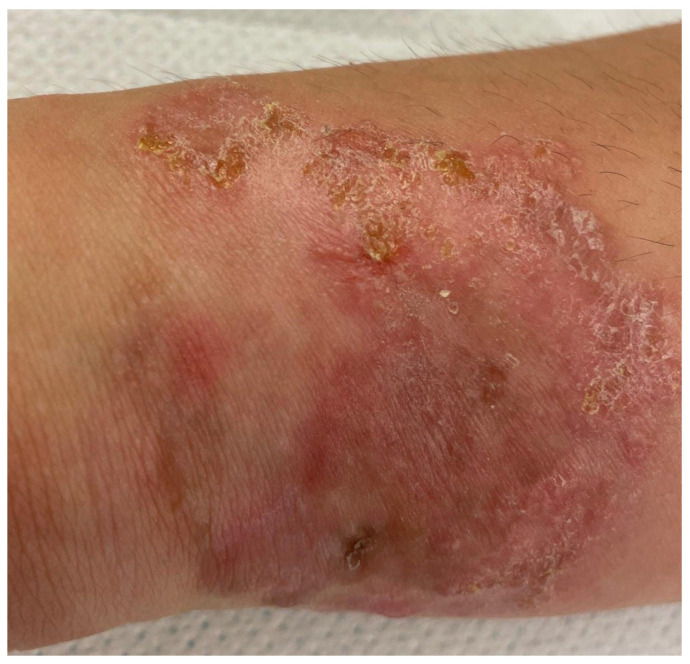
Figured lesion with clear margins and irregular edges, predominantly bright red erythema with the presence of crusty, scaly elements.
